# Soft X-ray varied-line-spacing gratings fabricated by near-field holography using an electron beam lithography-written phase mask

**DOI:** 10.1107/S1600577519008245

**Published:** 2019-08-16

**Authors:** Dakui Lin, Zhengkun Liu, Kay Dietrich, Andréy Sokolov, Mewael Giday Sertsu, Hongjun Zhou, Tonglin Huo, Stefanie Kroker, Huoyao Chen, Keqiang Qiu, Xiangdong Xu, Franz Schäfers, Ying Liu, Ernst-Bernhard Kley, Yilin Hong

**Affiliations:** a National Synchrotron Radiation Laboratory, University of Science and Technology of China, Hezuohua South Road 42, Hefei 230029, People’s Republic of China; b Institut für Angewandte Physik, Friedrich-Schiller-Universität Jena, Max-Wien-Platz 1, 07743 Jena, Germany; c Department for Nanometre Optics and Technology, Helmholtz-Zentrum Berlin für Materialien und Energie, Albert-Einstein-Strasse 15, 12489 Berlin, Germany; d Laboratory for Emerging Nanometrology, Technische Universität Braunschweig, Pockelsstrasse 14, 38106 Braunschweig, Germany; e Physikalisch-Technische Bundesanstalt, Bundesallee 100, 38116 Braunschweig, Germany

**Keywords:** near-field holography, electron beam lithography, fabrication, soft X-ray varied-line-spacing grating, spectrometer

## Abstract

A fabrication method comprising near-field holography (NFH) with an electron beam lithography (EBL)-written phase mask was developed to fabricate soft X-ray varied-line-spacing gratings (VLSGs). An EBL-written phase mask with an area of 52 mm × 30 mm and a central line density greater than 3000 lines mm^−1^ was used. The introduction of the EBL-written phase mask substantially simplified the NFH optics for pattern transfer. The characterization of the groove density distribution and diffraction efficiency of the fabricated VLSGs indicates that the EBL–NFH method is feasible and promising for achieving high-accuracy groove density distributions with corresponding image properties. Vertical stray light is suppressed in the soft X-ray spectral range.

## Abstract 2

A method comprising near-field holography with an electron beam lithography-written phase mask was developed herein. Soft X-ray varied-line-spacing gratings with a central groove density greater than 3000 lines mm^−1^ were fabricated using this method.

## 1. Introduction   

Soft X-ray spectrometers operating with photon energies of 100 eV to 1000 eV using plane (Hague *et al.*, 2005 ▸) or spherical (Koike & Namioka, 1997 ▸; Namioka & Koike, 1995 ▸) varied-line-spacing gratings (VLSGs) are being increasingly applied in synchrotron radiation (Chuang *et al.*, 2005 ▸; Ghiringhelli *et al.*, 2006 ▸; Osborn & Callcott, 1995 ▸; Qiao *et al.*, 2017 ▸; Strocov, 2010 ▸; Strocov *et al.*, 2011 ▸; Yin *et al.*, 2015 ▸; Wernet *et al.*, 2015 ▸) and plasma diagnosis (Dinh *et al.*, 2016 ▸; Poletto *et al.*, 2001 ▸; Shatokhin *et al.*, 2018 ▸). VLSGs are indispensable for soft X-ray spectrometers. Either a spherical VLSG or a combination of a spherical mirror with a plane VLSG can produce flat-field images. In this way, VLSGs enable the convenient recording of spectral images by flat detectors, for which soft X-ray VLSGs (SXVLSGs; XVLSGs for brevity in the following) with high central density up to 3500 lines mm^−1^ (Strocov, 2010 ▸; Strocov *et al.*, 2011 ▸), high groove density distribution precision (Chowdhuri *et al.*, 2007 ▸; Voronov *et al.*, 2017 ▸) and low stray light are strongly required.

Typical fabrication methods for XVLSGs include holographic lithography (Koike & Namioka, 1997 ▸; Namioka & Koike, 1995 ▸) and mechanical ruling (Harada & Kita, 1980 ▸; Kita *et al.*, 1983 ▸; Koike & Namioka, 1997 ▸). Holographic lithography has the advantages of low stray light and low high-order harmonics (Chowdhuri *et al.*, 2007 ▸; Yamazaki *et al.*, 1999 ▸). Mechanical ruling is more flexible regarding the groove density distribution than holographic lithography. As a typical nanofabrication technique, electron beam lithography (EBL) can write high-resolution patterns with an address grid of 0.1 nm and a flexible groove density distribution within a reasonable time frame (Harzendorf *et al.*, 2014 ▸). Therefore, two hybrid fabrication methods for XVLSGs have recently been developed based on EBL to overcome the limitations of previous methods and to meet the high-precision fabrication requirements of VLSGs. In each case, EBL is utilized to fabricate a mask. A subsequent pattern transfer process is necessary for efficient and economical use of the mask. In one of the methods, EBL was combined with nanoimprinting (Voronov *et al.*, 2017 ▸) to fabricate a 900 lines mm^−1^ XVLSG for a fluorescence spectrometer. In the other method, EBL is combined with near-field holography (NFH) (Kley & Clausnitzer, 2003 ▸; Lin *et al.*, 2018a ▸), which can also be called interference holography with an EBL-written mask. Without extra plasma etching (Voronov *et al.*, 2017 ▸), the duty cycle of XVLSGs can be tailored in this method by optimizing exposure and development during NFH. This results in an NFH process that is substantially simpler than nanoimprinting. Furthermore, the NFH method can be extended to the fabrication of blazed gratings (Lin & Li, 2008 ▸). For both hybrid methods, the stitching errors in the EBL-written masks can lead to stray light or the Rowland ghost of XVLSGs.

This study focuses on the fabrication of XVLSGs using the EBL–NFH method. Section 2 briefly introduces the specifications of the XVLSG with a central line density of 3600 lines mm^−1^ as the starting point of the study. Section 3 describes the static and dynamic NFH setups and the corresponding groove density distributions of the phase masks. The same section also introduces the aims and sample considerations based on static and dynamic NFH setups. The transfer of groove density distribution from an EBL-written phase mask to an XVLSG and stray light suppression are two key issues to resolve in the EBL–NFH method. To show that we have resolved these, we demonstrate the characterization of the groove density distribution and the diffraction efficiencies of the fabricated gratings and discuss the imaging properties and stray light suppression in Section 4. Finally, Section 5 summarizes the key results and future work.

## 2. Specifications of a soft X-ray VLSG   

**Figure 1:**
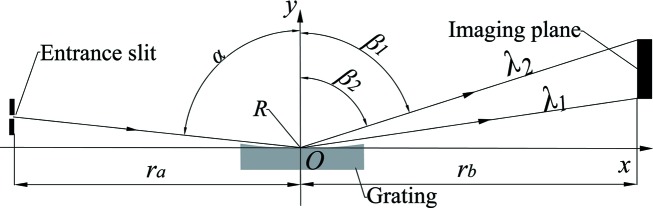
Schematics of a soft X-ray spectrometer based on a spherical VLSG.

The starting point of this study is an XVLSG based on a widely used geometry typical for flat-field spectrometers (Chowdhuri *et al.*, 2007 ▸; Imazono *et al.* 2007 ▸; Kita *et al.*, 1983 ▸). The gratings used in these spectrometers have a central density up to 2400 lines mm^−1^. To enhance the resolving power, we designed an XVLSG with a central line density of 3600 lines mm^−1^. As shown in Fig. 1 ▸, the length of the entrance (exit) arm *r*_a_ (*r*_b_) corresponds to the distance between the grating centre and the entrance slit (detector plane). These lengths were 237 mm and 235 mm, respectively. The spectral range of the spectrometer was designed over the range from 0.3 nm (λ_1_) to 0.6 nm (λ_2_). To operate at angle of incidence α of 88.65°, the diffraction angles β_1_ and β_2_ of the grating centre (3600 lines mm^−1^) at wavelengths of 0.3 nm and 0.6 nm were 87.01° and 85.99°, respectively. The radius of curvature *R* of the XVLSG substrate was 12354 mm. The groove density distribution *n*_opt_(*x*) [equation (1) of the XVLSG, where *x* is the position along the grating vector with units of mm] was optimized with the above-mentioned specifications according to the ray-tracing method (Harada & Kita, 1980 ▸; Harada *et al.*, 1999 ▸) and the program was written in MATLAB.
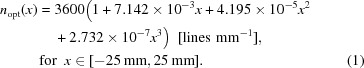



## 3. Near-field holography setups   

### 3.1. Static NFH setup   

**Figure 2:**
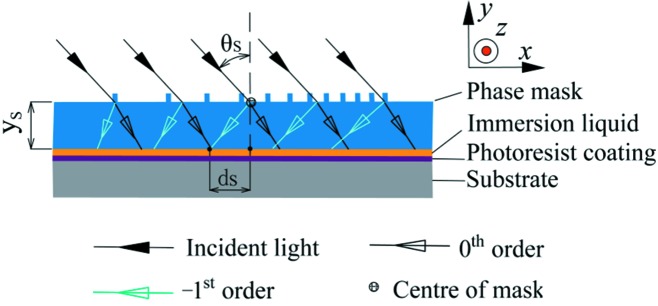
Schematic of the SNFH with an EBL-written phase mask for XVLSG fabrication.

Fig. 2 ▸ shows a conventional NFH setup denoted as the static NFH (SNFH) (Amako & Sawaki, 2012 ▸; Li *et al.*, 2016 ▸). The unstructured surface of the phase mask faces the photoresist layer on a fused silica grating substrate (refractive index *n* = 1.482–325 nm). A layer of immersion liquid (or refractive index liquid) is used to fill the gap between the phase mask and the grating substrate. The thickness *y*_S_ of the phase mask is 6 mm and the wavefront of the mask is 0.184 ± 0.022 λ′ (λ′ = 632 nm). Upon illumination of the phase mask at λ = 325 nm and an angle of incidence θ_S_ = 37.5°, two diffraction orders appear in the transmittance indicated by either 0th- or −1st-order diffraction, both of which cause interference. The interference pattern is recorded by the photoresist layer and a photoresist grating mask is made ready for an XVLSG.

The groove density distribution *n*_pm_S_(*x*) of the phase mask for SNFH is dependent on the groove density distribution *n*_opt_(*x*) of the XVLSG and the NFH geometry (λ, θ_S_ and thickness of the phase mask *y*_S_). The thickness of the immersion liquid is approximately 60 µm and can be omitted during the simulation of the groove density distribution *n*_pm_S_(*x*). As shown in Fig. 2 ▸, the groove density at a certain position (*x*) on the phase mask is transferred to the grating substrate with a displacement of *d*_S_(*x*) along the (−*x*)-axis as follows (Lin *et al.* 2018 ▸ *b*),
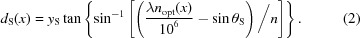
With *d*_S_(*x*) and *n*_opt_(*x*), the groove density of the phase mask at position *x* can be calculated point by point. The fitted 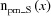 can then be expressed as follows,
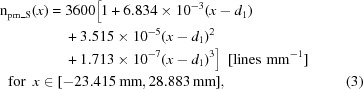
where *d*_1_ = *d*_S_(0) = 2.456 mm. This value corresponds to the displacement at the grating centre with a line density of 3600 lines mm^−1^.

The groove density distribution of an XVLSG plays an important role in determining the imaging properties of a flat-field spectrometer. When it is optimized, spectral images can form on a flat-field detector plane. We characterized the groove density of the photoresist grating mask *n*_meas_(*x*), which was further utilized to simulate the imaging properties of the XVLSGs in order to demonstrate the feasibility of NFH in transferring groove density distributions. We utilized the photoresist grating mask instead of the final XVLSG for characterization to achieve a sufficiently high diffraction intensity at a wavelength of 360 nm.

### 3.2. Dynamic NFH setup   

Stitching errors, which provoke parasitic diffraction orders known as Rowland ghosts, are inherently associated with EBL (Heusinger *et al.*, 2016 ▸, 2017 ▸). A dynamic NFH (DNFH) (Lin *et al.*, 2018*a* ▸) was developed to suppress the stitching errors of the phase mask.

**Figure 3:**
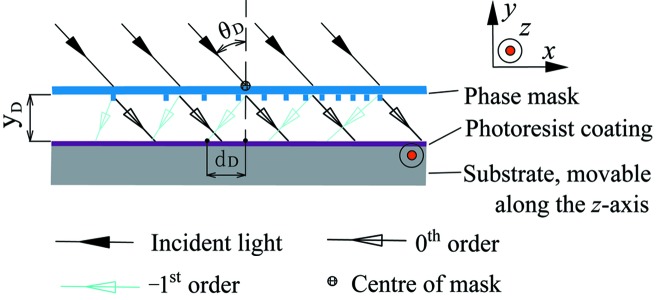
Schematic of the dynamic NFH with an EBL-written phase mask for XVLSG fabrication. The grating substrate can be displaced along the *z* axis during dynamic exposure.

Fig. 3 ▸ presents a simplified sketch of the developed DNFH setup for generating the photoresist pattern of an XVLSG. The DNFH setup differs from the SNFH in the following ways: the phase mask is placed at a distance *y*_D_ = 3 mm in front of a photoresist-coated fused silica grating substrate; the gap between the phase mask and the substrate is filled with air and the structured surface of the phase mask faces the photoresist layer of the grating substrate. The phase mask is illuminated at a wavelength of 325 nm and an angle of incidence θ_D_ = 35.5°. Correspondingly, the displacement of *d*_D_(*x*) of the light at the −1st order at a certain *x* can be expressed as follows,
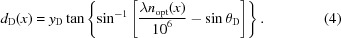
Similar to that of the SNFH, the fitted 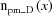 of a phase mask for the DNFH can be expressed as
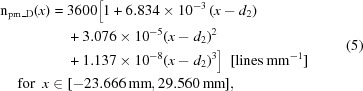
where *d*_2_ = *d*_D_(0) = 2.188 mm. This value corresponds to the displacement at the grating centre with a line density of 3600 lines mm^−1^. Therefore, the groove density distributions of the phase masks for SNFH and DNFH should, in principle, be different.

A movable substrate during NFH is the main feature of the DNFH (*i.e.* the substrate is moved along the *z* axis parallel to the grating ridges during the exposure). Subsequently, after exposure and development, the obtained photoresist grating of the XVLSG is proportionally transferred by reactive ion beam etching and homogeneously coated with gold (He *et al.*, 2011 ▸).

A successful demonstration of this approach was preliminarily verified by photoresist gratings at a wavelength of 441.6 nm (Lin *et al.*, 2018*a* ▸). However, the effect of the DNFH on the diffraction efficiency and stray light in the soft X-ray spectral range is still missing. Therefore, the in-plane and off-plane diffraction efficiencies (Seely *et al.*, 2006 ▸) of the fabricated gratings were measured and compared in the spectral range 1–6 nm to characterize this effect. Note that only the phase mask for the SNFH was available herein because the DNFH was developed from the SNFH. Hence, the phase mask for the SNFH was employed during the DNFH. Correspondingly, the central line density of the fabricated gratings was approximately 3340 lines mm^−1^, which deviated from the optimized 3600 lines mm^−1^. Samples A and B were exposed with the DNFH setup. The grating substrates were static (Sample A) and dynamic (Sample B) during exposure.

Note that the range of grating line densities that may be fabricated using this technology depends on the laser wavelength, incidence angle and NFH configuration. The available grating periods, ‘period’, with the use of this method need to fulfil the following equations:
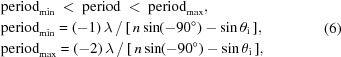
where *n* is the refractive index of the diffraction medium and θ_i_ is the incidence angle. The available minimal and maximal periods, period_min_ and period_max_, can be calculated according to the grating equation (Harvey & Vernold, 1998 ▸). The sign of θ_i_ is ‘+’ if the incidence ray is projected along the (+*x*) axis, and ‘−’ if the projection is along the (−*x*) axis. Equation (6) suggests that the available minimal period, period_min_, should be large enough to keep the absolute diffraction angle of the −1st order smaller than 90°, and the available maximal period, period_max_, should be small enough to keep the absolute diffraction angle of the −2nd order at an oblique incidence of more than 90°. Taking the DNFH configuration as an example, if λ = 325 nm, θ_i_ = 35° and *n* = 1, the grating period that can be transferred via DNFH ranges from 207 nm to 413 nm.

To achieve balanced diffraction efficiencies at the 0th and −1st orders, the groove profile parameters of the phase mask were optimized according to the NFH geometry (Gâté *et al.*, 2013 ▸; Kley & Clausnitzer, 2003 ▸) using rigorous coupled wave analysis (RCWA) (Moharam *et al.*, 1995a ▸, ▸ *b*). The phase mask was optimized with a duty cycle of 0.52 ± 0.02 and a depth of 250 ± 5 nm at a central line density of 3600 lines mm^−1^. The optimized diffraction efficiencies of the 0th and −1st orders were approximately 48 ± 2%.

## 4. Results and discussion   

### 4.1. Imaging property simulation of the fabricated VLSG   

**Figure 4:**
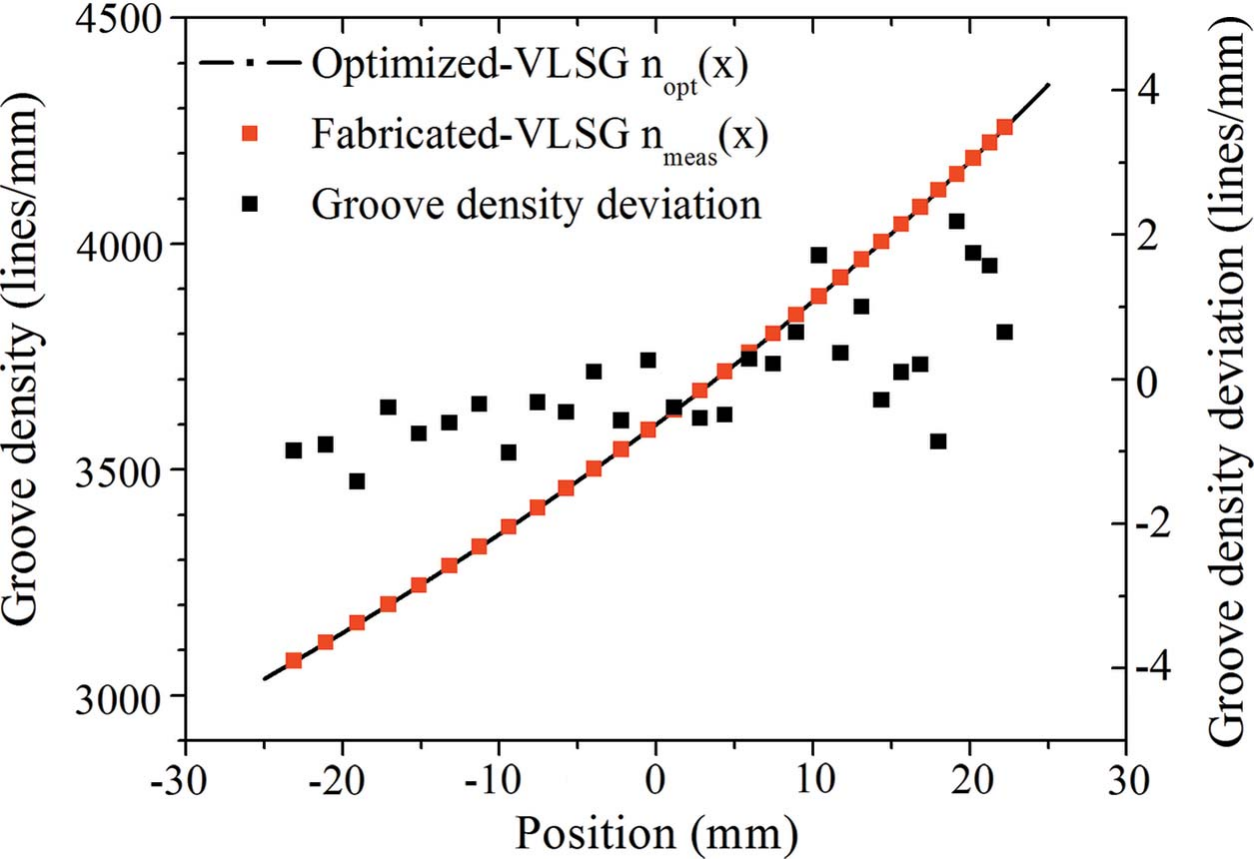
Optimized and measured groove density distribution of an XVLSG and a fabricated VLSG. The quantity *n*_opt_(*x*) is the optimized groove density distribution of the XVLSG and *n*_meas_(*x*) denotes the measured groove density distribution of the fabricated VLSG.

The groove densities of the fabricated grating were measured (red blocks in Fig. 4 ▸) at several positions along the grating vector (the *x* axis) by the optical diffraction method under the Littrow condition (Hu *et al.*, 2004 ▸) using a laser source with a wavelength of 360 nm.

**Figure 5:**
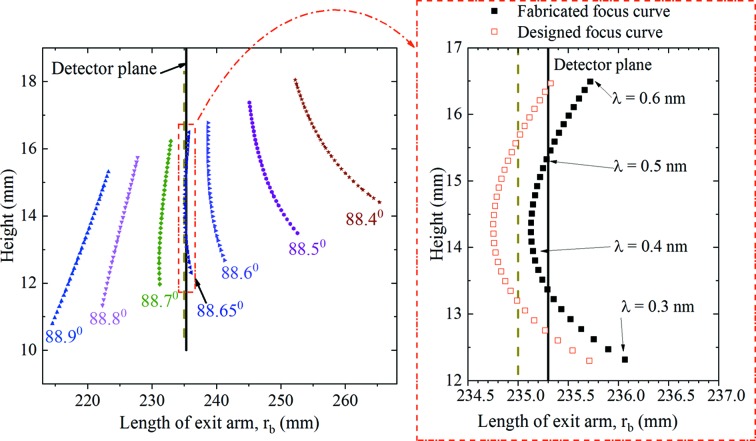
Focal curves calculated with the groove density distribution *n*_meas_(*x*) of the fabricated grating at different incidence angles. The dashed and solid vertical lines indicate the optimized detector positions calculated with *n*_opt_(*x*) of the XVLSG and *n*_meas_(*x*) of the fabricated VLSG, respectively. The empty red and solid black blocks are the simulated focus curves of the VLSGs with *n*_opt_(*x*) and *n*_meas_(*x*), respectively.

The measured groove densities were fitted as the groove density distribution *n*_meas_(*x*) [equation (7)] of the fabricated grating for comparison with the optimized *n*_opt_(*x*) [black line in Fig. 4 ▸ and equation (1)],
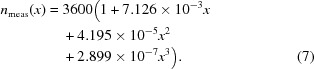
The relative measurement error of the groove density distribution is ±7 × 10^−5^. The groove density distributions of the optimized and measured gratings are in good agreement. The maximum deviation is 2.2 lines mm^−1^ at position *x* = 19.19 mm. With the groove density distribution *n*_meas_(*x*) of the fabricated grating, the dependence of the focus point on the height at the detector plane and angle of incidence was calculated at several wavelengths by the ray-tracing method as shown in Fig. 5 ▸. Note that the photoresist grating was fabricated on a plane substrate. We corrected the groove density distribution of the plane substrate *n*_meas_(*x*) to that of a spherical substrate *n*_cor_(*x*) with an *R* of 12354 mm. For the spherical substrate, the coefficients of *x* ^2^ and *x* ^3^ of the groove density distribution *n*_cor_(*x*) are reduced by 1.6 × 10^−7^ and 4.1 × 10^−9^, respectively. Our simulation indicates that the same results of imaging properties can be achieved with *n*_meas_(*x*) and *n*_cor_(*x*). The radius of the spherical substrate is very large; hence, the groove density distributions of a plane and the spherical substrates have the same influence on the imaging properties. Therefore, utilizing *n*_meas_(*x*) of a plane substrate for the following simulation is reasonable. The angle of incidence is an important parameter affecting the focus curve. If the incidence angle was not 88.65°, it could cause significant defocus, inclination or curvature of the focus curve. As indicated in the inset in Fig. 5 ▸, the focus curves of both the optimized (red empty blocks) and fabricated (black solid blocks) gratings are within the defocus length less than 1 mm. The maximal and minimal lengths of defocus of the optimized and fabricated gratings are 0.5 mm and −0.3 mm and 0.6 mm and −0.2 mm at the wavelengths of 0.3 nm and 0.45 nm, respectively. The minimum focus deviation is not at 235 mm (red empty blocks in the inset of Fig. 5 ▸) anymore because of the difference in the density distribution between the specified and real cases. However, this can be corrected by slightly increasing the arm length to 235.3 mm (black solid blocks in the inset of Fig. 5 ▸).

**Figure 6:**
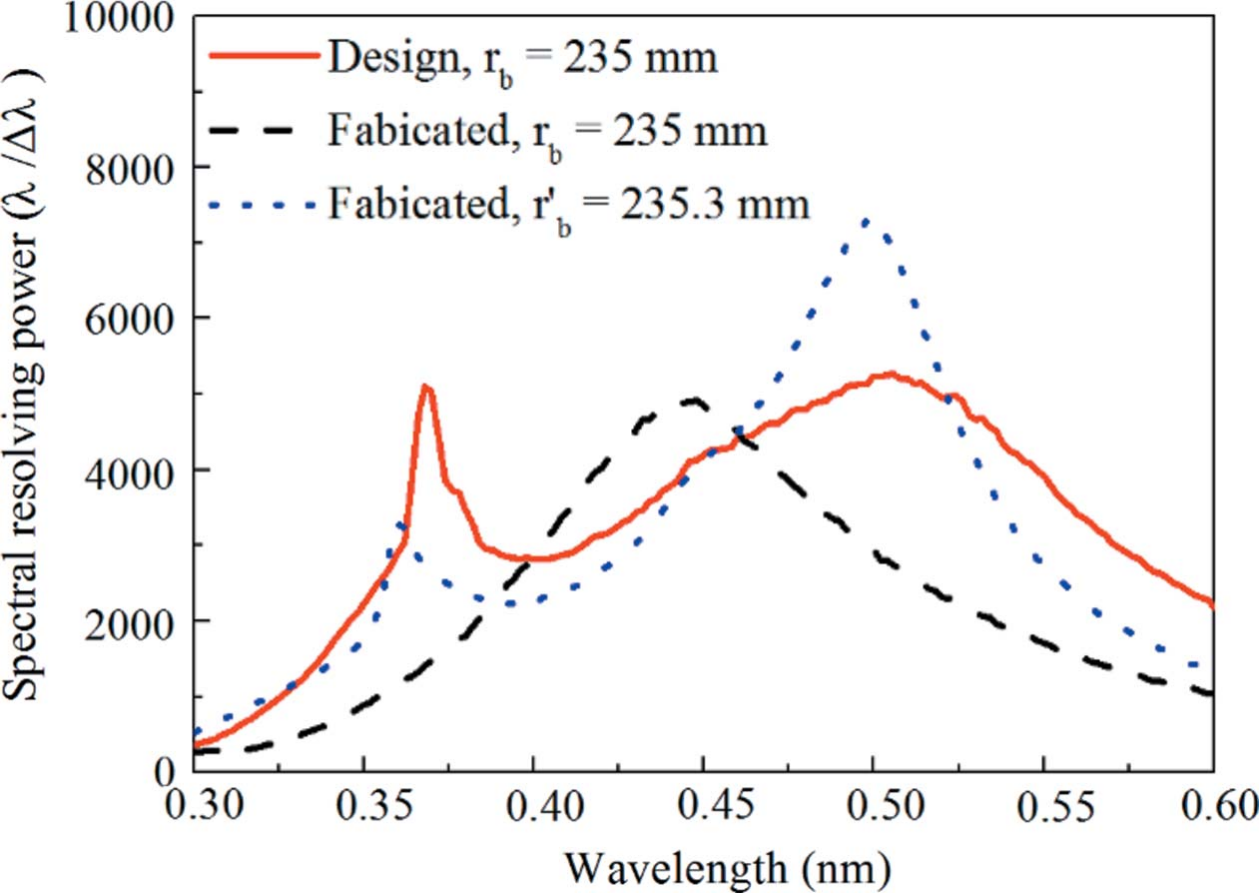
Simulated spectral resolving power of the optimized and fabricated gratings at the optimized and modified imaging planes, respectively. *r*_b_ is the optimized length of the exit arm, *r*_b_′ is the optimized length of the exit arm according to the groove density distribution of the fabricated grating.

Fig. 6 ▸ demonstrates the simulated spectral resolving power (λ/Δλ) (Löchel *et al.*, 2015 ▸) of the designed and fabricated XVLSG. We assumed that the incident light source is an ideal point source; the charge coupled detector has a pixel size of 0.02 µm × 0.02 µm and the total number of rays is 20 000 000 (Chuang *et al.*, 2005 ▸). It shows the upper limit of the spectral resolving power that can be achieved. The non-monotonic wavelength of the spectral resolving power stems from the focus curve on the detector plane (Fig. 5 ▸). The less defocused the focus position is, the higher the spectral resolving power is. The curves of the designed grating (*r*_b_ = 235 mm, red line) and the fabricated grating (*r*_b_′ = 235.3 mm, black line) present two peaks at the wavelengths of 0.37 nm and 0.51 nm, and 0.36 nm and 0.50 nm, respectively. The two peaks in each curve indicate that the spectal images are well focused on the detector plane at the two corresponding wavelengths. In contrast, the curve of the fabricated grating (*r*_b_ = 235 mm, blue line) only shows one peak at 0.45 nm. Hence, the simulated spectral resolving powers (Fig. 6 ▸) and the focal curves (Fig. 5 ▸) agree well. Note that the maximum resolving power of the fabricated grating (*r*_b_ = 235 mm) at a wavelength of 0.45 nm was even higher than that of the designed grating because of the smallest distance between the focal position and the detector plane. However, the spectral resolving power of the fabricated grating (*r*_b_ = 235 mm) was degraded at its two ends, which could be attributed to the large defocus at the two ends. To overcome this, it is suggested that a slight deviation in the detector ensures a focus curve with a slight defocus less than 1 mm (solid line at the *r*_b_′ = 235.3 mm position in Fig. 5 ▸). Correspondingly, the overall spectral resolving power of the fabricated grating significantly improved across the entire spectral range when the length of the exit arm is changed from 235 mm (black line in Fig. 6 ▸) to 235.3 mm (blue line in Fig. 6 ▸). The simulation results indicate that an error in the groove density of the fabricated grating upon the imaging properties can be compensated for by adjusting the axial detector position. From these simulations we conclude that the groove density of the fabricated grating could be effectively transferred from the phase mask by NFH.

### 4.2. Diffraction efficiency characterizations   

**Figure 7:**
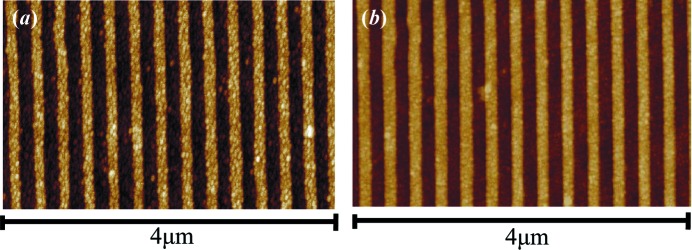
AFM images of the gold-coated VLSGs fabricated by (*a*) SNFH and (*b*) DNFH.

Fig. 7 ▸ shows the AFM images of the gold-coated XVLSGs fabricated by SNFH (Sample A) and DNFH (Sample B). Sample B has a lower line edge roughness and a more uniform line width than Sample A. These features were the same as those of the photoresist gratings demonstrated in our previous paper (Lin *et al.*, 2018 ▸ *a*). Table 1 ▸ summarizes the groove parameters of the fabricated gratings based on SNFH (Sample A) and DNFH (Sample B). The in-plane and off-plane diffraction efficiencies of the fabricated gratings were characterized to compare the effects of the DNFH and SNFH exposure modes.

**Figure 8:**
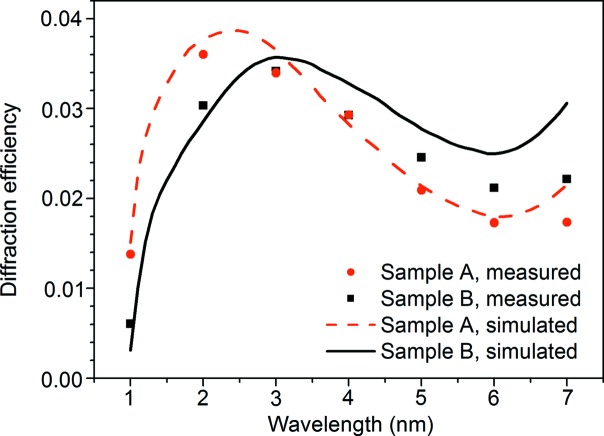
Simulated and measured −1st-order in-plane efficiencies of the two fabricated XVLSGs as functions of the wavelengths at an incidence angle of 87°.

Fig. 8 ▸ presents the simulated and measured in-plane efficiencies [the projection direction of the incident light on the grating surface was parallel to the grating vector direction (*x* axis)] of the two XVLSGs at the −1st order. The diffraction efficiencies of the soft X-ray gratings were characterized at wavelengths of 1–6 nm at the Optics Beamline/Reflectometer at Helmholtz-Zentrum Berlin (Schäfers *et al.*, 2016 ▸; Schäfers & Sokolov, 2016 ▸; Sokolov *et al.*, 2016 ▸). The Optics Beamline can supply working energy from 10 eV to 2000 eV with high spectral purity. A multilayer is necessary to increase the grating diffraction efficiency at such a short X-ray range (Choueikani *et al.*, 2014 ▸; Imazono *et al.*, 2007 ▸, 2013 ▸, 2018 ▸; Koike *et al.*, 2009 ▸; Senf *et al.*, 2016 ▸; Vantelon *et al.*, 2016 ▸; Yang *et al.*, 2017 ▸). However, the multilayer was not the focus of this study; hence gold was used to coat the fabricated gratings. Correspondingly, the diffraction efficiencies of the gratings were measured for the 1–6 nm spectral range instead of the 0.3–0.6 nm range. A comparison of the measured and simulated diffraction efficiencies verifies the design and fabrication. We can infer the design and performance of the diffraction efficiency in the 0.3–0.6 nm range. The angle of light incident onto the grating was set to 87° instead of the designed angle of 88.65° to ensure that all the diffracted light was received by the detector. The spot size of the incident light was 4 mm × 0.3 mm, which corresponded to a line density range from 3342 lines mm^−1^ to 3443 lines mm^−1^ for the grating (with an average of ∼3390 lines mm^−1^). The RCWA simulation indicates that the diffraction efficiencies for each groove density at the −1st order are 3.7% and 3.6%, respectively. These results further imply that the relative deviation of the diffraction efficiency caused by the spatial variation in the groove density is approximately 5%. A central groove density of 3389 lines mm^−1^ was utilized, and the effect of groove density on the diffraction efficiency was neglected to simplify the simulation.

**Figure 9:**
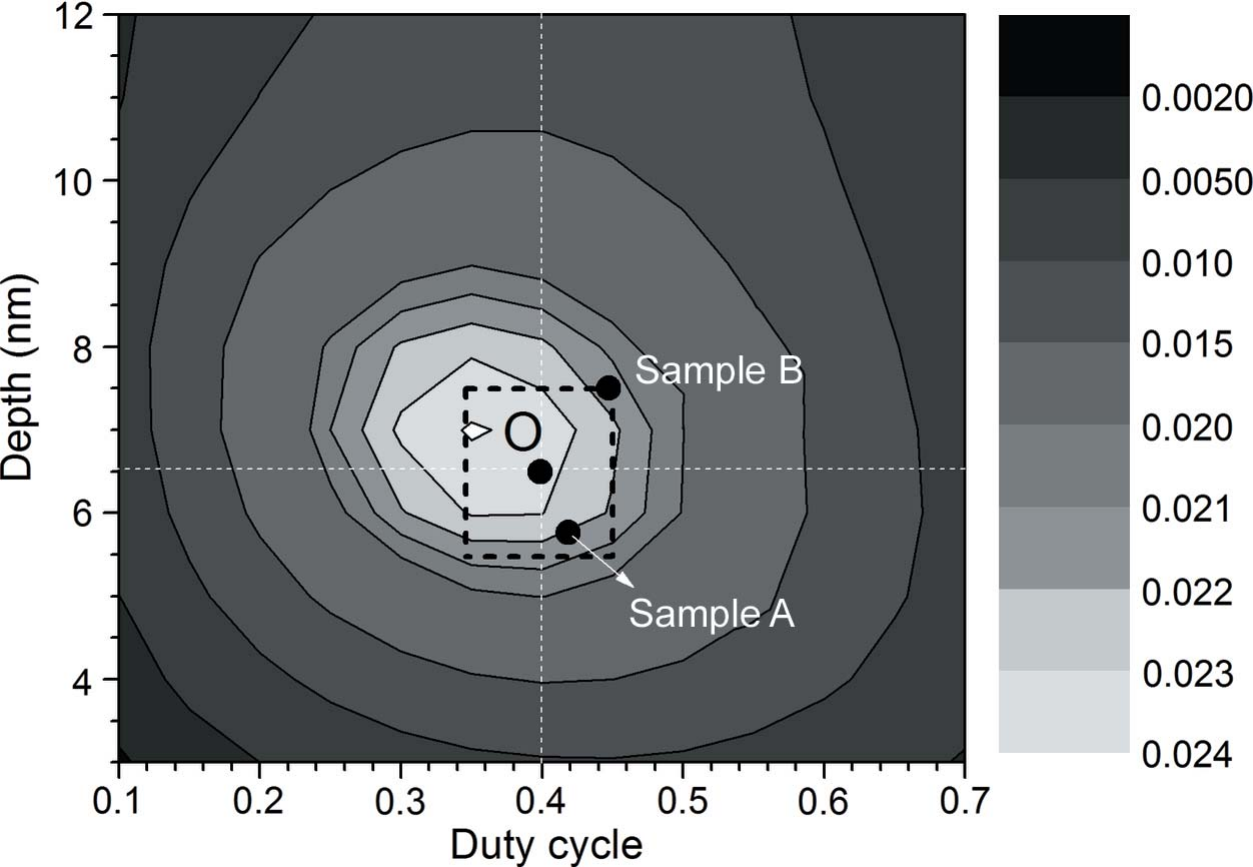
Optimized and fabricated profiles of the gratings. The simulation was performed as functions of depth and duty cycle at a wavelength of 3 nm and an incidence angle of 88.65°. This implies that grating efficiencies would be improved if both the depth and duty cycle of the two samples approach the centre (point O) with a depth of 6.5 nm and duty cycle of 0.40.

Table 1 ▸ summarizes the measured groove parameters for both samples. The RCWA simulation was then performed. As presented in Fig. 8 ▸, the measured efficiency at a wavelength of 1 nm was less than 1% and the magnitude of its diffraction intensity approached that of the detector noise. Therefore, the efficiency at 1 nm was excluded from the analysis. The measured −1st order efficiencies of both gratings were in good agreement with the simulated efficiency. The maximal deviations of the measured efficiencies of both gratings were ∼19% (Sample A) and ∼28% (Sample B) less than those of the calculated efficiencies. The maximal measured efficiencies of both samples are ∼3.5%, which is ∼95% of the simulated efficiencies at wavelengths of 3 nm and 2 nm. Fig. 9 ▸ shows the optimized in-plane efficiency as functions of depth and duty cycle at a wavelength of 3 nm and an incidence angle of 88.65°. This indicates that the optimized groove profile is with a depth of 6.5 ± 1.0 nm and duty cycle of 0.40 ± 0.05 (black dashed block in Fig. 9 ▸). Similarily, for gratings in the range 0.3–0.6 nm, the optimized groove parameter is with a depth of 3 ± 0.5 nm and duty cycle of 0.45 ± 0.10. Moreover, the fabricated grating profiles are also illustrated as samples A and B. The results indicate that the grating efficiency can be improved if the groove parameters approach a depth of 6.5 nm and duty cycle of 0.40 (point O in Fig. 9 ▸). Additionally, the efficiency difference between the two gratings is mainly attributed to the deviations in their groove profiles. Therefore, no significantly different effects were observed for DNFH and SNFH during the in-plane efficiency measurement.

**Figure 10:**
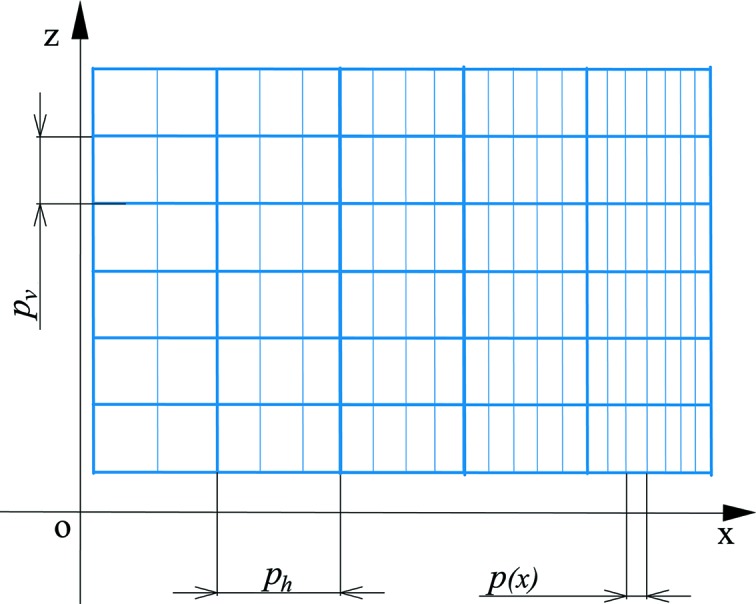
Sketch of the primary grating patterns [*i.e.* patterns for an XVLSG, the grating vector of which is along the *x* axis; *p*(*x*) is the grating period at position *x*] and the horizontal and vertical stitching errors (periods indicated as *p*_h_ and *p*_v_ and grating vectors present along the *x* and *z* axes, respectively) on an EBL-written VLS phase mask.

**Figure 11:**
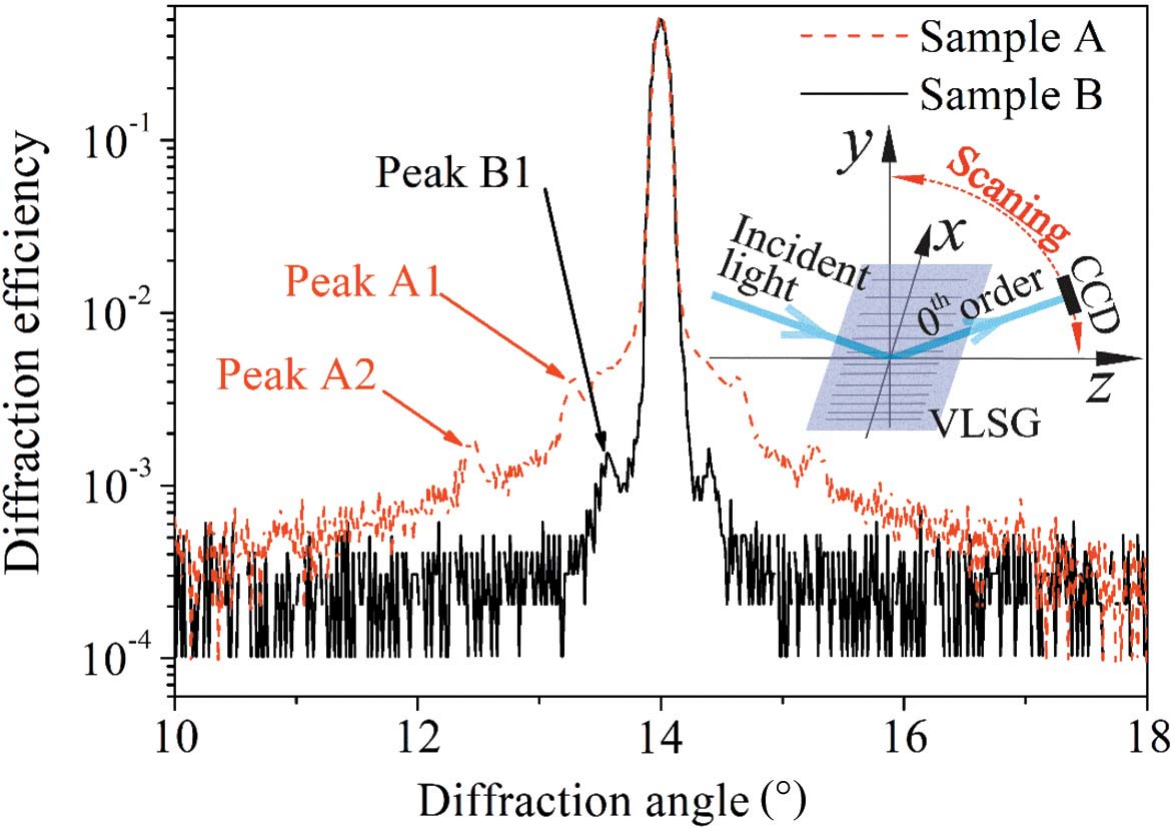
Measured off-plane efficiency angular spectra of the fabricated gratings (at a wavelength of 5 nm and an incidence angle of 83°) as a function of the diffraction angle.

We also investigated the effects of DNFH and SNFH on stray light. The stitching errors on an EBL-written phase mask with an area of 52 mm × 30 mm were inevitable (Heusinger *et al.*, 2016 ▸, 2017 ▸). Fig. 10 ▸ is a schematic of the primary grating patterns (*i.e.* patterns for the XVLSG and the horizontal and vertical stitching errors on a phase mask). The size of the horizontal and vertical stitching error or period is 625 µm and 35 µm, respectively (Heusinger *et al.*, 2017 ▸; Lin *et al.*, 2018*a* ▸). The vertical stitching error of the phase mask was smoothed during DNFH (Lin *et al.*, 2018*a* ▸). To observe the diffraction of the vertical stitching error, the projection direction of the incident light should be parallel to the grating vector of the vertical stitching error (along the *z* axis in Fig. 10 ▸), which corresponds to measuring the off-plane diffraction efficiencies of the fabricated XVLSGs. Fig. 11 ▸ indicates the off-plane diffraction efficiency angular spectra at a wavelength of 5 nm and an incident angle of 83° and compares the effects of DNFH and SNFH on the stray light of gratings. The vertical Rowland ghosts of the fabricated gratings were characterized at the spectral radiation standard and metrology beamline (BL08B) of the National Synchrotron Radiation Laboratory of China (He *et al.*, 2011 ▸). As indicated in Fig. 11 ▸, the peak efficiencies of the two curves were similar, illustrating that DNFH and SNFH had similar effects on the main efficiency of both samples. However, obvious differences can be found in both curves regarding their stray light. Firstly, the stray light level of Sample B is approximately an order of magnitude less than that of Sample A. Therefore, the DNFH suppressed the stray light level of the grating. Secondly, several small peaks symmetrically distributed beside each large peak are visible. A higher number of small peaks were observed in Sample A (peaks A1 and A2) than Sample B (peak B1). These small peaks can be attributed to the diffraction of the vertical stitching error from the EBL-written VLS mask, with a period of 35 µm. The number and the intensity of small peaks occurring as a result of the vertical stitching error was reduced through the introduction of the DNFH. Subsequently, the vertical Rowland ghost of the fabricated VLSG was suppressed.

## 5. Conclusions   

A fabrication method consisting of EBL and NFH was developed for XVLSGs for application in high-resolution X-ray spectrometers. The EBL–NFH method exhibits the advantages of both EBL (high line density and high-accuracy groove density distribution) and NFH (fast transfer of high-resolution VLSG patterns and flexibility in tailoring groove profile parameters of XVLSGs).

Plane VLSGs with a central density larger than 3000 lines mm^−1^ were fabricated using this method. The characterization indicated that the groove density distributions and diffraction efficiencies at a wavelength range of 1–6 nm of the fabricated and designed gratings were in good agreement, which is promising for good imaging properties. Moreover, the proposed DNFH can significantly suppress the vertical Rowland ghosts of XVLSGs, which is important for gratings working at X-ray spectral ranges.

Therefore, the EBL–NFH method demonstrates a promising option for the realization of VLSGs for high-end soft X-ray spectrometers. Future research should extend the implementation of the method from planar to spherical VLSGs and upgrade the Au coating to multilayers.

## Figures and Tables

**Table 1:** Measured parameters of the grating samples

	Line density (lines mm^−1^)	Duty cycle	Depth (nm)
Sample A	3387.41 ± 1.89	0.41 ± 0.04	5.6 ± 0.8
Sample B	3389.52 ± 1.69	0.45 ± 0.02	7.4 ± 0.5
